# Genetic Variants on Chromosome 1q41 Influence Ocular Axial Length and High Myopia

**DOI:** 10.1371/journal.pgen.1002753

**Published:** 2012-06-07

**Authors:** Qiao Fan, Veluchamy A. Barathi, Ching-Yu Cheng, Xin Zhou, Akira Meguro, Isao Nakata, Chiea-Chuen Khor, Liang-Kee Goh, Yi-Ju Li, Wan'e Lim, Candice E. H. Ho, Felicia Hawthorne, Yingfeng Zheng, Daniel Chua, Hidetoshi Inoko, Kenji Yamashiro, Kyoko Ohno-Matsui, Keitaro Matsuo, Fumihiko Matsuda, Eranga Vithana, Mark Seielstad, Nobuhisa Mizuki, Roger W. Beuerman, E.-Shyong Tai, Nagahisa Yoshimura, Tin Aung, Terri L. Young, Tien-Yin Wong, Yik-Ying Teo, Seang-Mei Saw

**Affiliations:** 1 Saw Swee Hock School of Public Health, National University of Singapore, Singapore, Singapore; 2 Singapore Eye Research Institute, Singapore National Eye Centre, Singapore, Singapore; 3 Department of Ophthalmology, National University of Singapore, Singapore, Singapore; 4 Department of Ophthalmology, Yokohama City University School of Medicine, Yokohama, Japan; 5 Department of Ophthalmology, Kyoto University Graduate School of Medicine, Kyoto, Japan; 6 Center for Genomic Medicine and Inserm U.852, Kyoto University Graduate School of Medicine, Kyoto, Japan; 7 Genome Institute of Singapore, Agency for Science, Technology, and Research, Singapore, Singapore; 8 Centre for Molecular Epidemiology, National University of Singapore, Singapore, Singapore; 9 Department of Pediatrics, National University of Singapore, Singapore, Singapore; 10 Duke–National University of Singapore Graduate Medical School, Singapore, Singapore; 11 Department of Medical Oncology, National Cancer Centre Singapore, Singapore, Singapore; 12 Department of Biostatistics and Bioinformatics, Duke University Medical School, Durham, North Carolina, United States of America; 13 Center for Human Genetics, Duke University Medical Center, Durham, North Carolina, United States of America; 14 Department of Molecular Life Science, Division of Molecular Medical Science and Molecular Medicine, Tokai University School of Medicine, Isehara, Japan; 15 Department of Ophthalmology and Visual Science, Graduate School of Medicine, Tokyo Medical and Dental University, Tokyo, Japan; 16 Division of Epidemiology and Prevention, Aichi Cancer Center Research Institute, Nagoya, Japan; 17 Institute for Human Genetics and Department of Laboratory Medicine, University of California San Francisco, San Francisco, California, United States of America; 18 Department of Medicine, National University of Singapore, Singapore, Singapore; 19 Centre for Eye Research Australia, University of Melbourne, Melbourne, Australia; 20 Graduate School for Integrative Science and Engineering, National University of Singapore, Singapore, Singapore; 21 Department of Statistics and Applied Probability, National University of Singapore, Singapore, Singapore; Harvard University, United States of America

## Abstract

As one of the leading causes of visual impairment and blindness, myopia poses a significant public health burden in Asia. The primary determinant of myopia is an elongated ocular axial length (AL). Here we report a meta-analysis of three genome-wide association studies on AL conducted in 1,860 Chinese adults, 929 Chinese children, and 2,155 Malay adults. We identified a genetic locus on chromosome 1q41 harboring the zinc-finger 11B pseudogene *ZC3H11B* showing genome-wide significant association with AL variation (rs4373767, β = −0.16 mm per minor allele, *P_meta_* = 2.69×10^−10^). The minor C allele of rs4373767 was also observed to significantly associate with decreased susceptibility to high myopia (per-allele odds ratio (OR) = 0.75, 95% CI: 0.68–0.84, *P_meta_* = 4.38×10^−7^) in 1,118 highly myopic cases and 5,433 controls. *ZC3H11B* and two neighboring genes *SLC30A10* and *LYPLAL1* were expressed in the human neural retina, retinal pigment epithelium, and sclera. In an experimental myopia mouse model, we observed significant alterations to gene and protein expression in the retina and sclera of the unilateral induced myopic eyes for the murine genes *ZC3H11A*, *SLC30A10*, and *LYPLAL1*. This supports the likely role of genetic variants at chromosome 1q41 in influencing AL variation and high myopia.

## Author Summary

Myopic individuals exhibit an increase in ocular axial length (AL). As a highly heritable ocular biometry of refractive error, identification of quantitative trait loci influencing AL variation would be valuable in informing the biological etiology of myopia. We have determined that a genetic locus on chromosome 1q41 containing zinc-finger pseudogene *ZC3H11B* is associated with AL and high myopia through a meta-analysis of three genome-wide association scans on AL in Chinese and Malays, with validation for high myopia association in two additional Japanese cohorts. In addition, variations in the expression of murine gene *ZC3H11A* and two neighboring genes *SLC30A10* and *LYPLAL1* in the retina and sclera in a myopic mouse model implicate the role of these genes in myopia onset. To our knowledge, this is the first genome-wide survey of single nucleotide polymorphism (SNP) variation of AL in Asians. Our results suggest that genetic variants at chromosome 1q41 have potential roles in both common and high myopia.

## Introduction

Myopia increases the risk of visual morbidity and poses a considerable public health and economic burden globally, especially in Asia, where the prevalence is significantly higher than other parts of the world [1]. Human myopia primarily results from an abnormal increase in ocular axial length (AL), the distance between the anterior and posterior poles of the eye globe, whereas the role of corneal curvature and lens thickness is minimal [2]. A 1 millimeter (mm) increase in AL is equivalent to a myopic shift of −2.00 to −3.00 diopters (D) with no corresponding changes in the optical power of the cornea and lens. High myopia, often defined as ocular spherical equivalent (SE) refraction below −6.00 D, is associated with an abnormally long AL, and this affects between 1% to 10% of the general population [3]. The degenerative changes in the retina and the choroid due to the excessive elongation of the globe are not prevented by optical correction and this subsequently increases the risk of visual morbidity through myopic maculopathy, choroidal neovascularization, retinal detachment and macular holes [4]. The active remodeling of the sclera, mediated by the signaling cascade initiated in the retina under visual input, has also been found to be critical in determining axial growth, and thus the refractive state of the eye [5].

Environmental factors such as the extent of near work, level of educational attainment and amount of outdoor activities have been documented to affect myopia development [6]. Evidence from family and twin studies has also supported a substantial genetic component in spherical refractive error and AL [7]–[9]. The heritability of the quantitative trait AL has been estimated to be as high as 94% comparable to that for SE (for a review, see [10]). Although linkage scans on pedigrees (myopia loci MYP1 to MYP18; see http://www.omim.org) and genome-wide association studies (GWAS) [11]–[16] have implicated several regions in the human genome as being significant for refractive error and myopia, no myopia genes have been consistently identified within or across different population groups. This scenario reflects the complexity in the disease architecture of myopia pathogenesis.

Genetic factors influencing AL and refraction appear to be at least partly shared, given previous literature from twin studies illustrating that at least half of the covariance between AL and refraction are due to common genetic factors [18]. The measurement of AL is more precise and less prone to errors compared to cycloplegic or non-cycloplegic assessments of refraction. As AL is an endophenotype for spherical refractive error, identifying genes that are responsible for AL variation provides insight into myopia predisposition and development. Presently there are only two genome-wide linkage studies performed in European descent populations that suggest the presence of AL quantitative trait loci (QTLs) on chromosomes 2p24 [19] and 5q (at 98 centimorgans) along with two classical myopia loci (MYP3 at 12q21 and MYP9 at 4q12) [20], and there are no reports of any genes that are indisputably confirmed to be associated with AL.

We thus performed a meta-analysis of three genome-wide surveys of AL in a total of 4,944 individuals in Asian populations comprising (i) Chinese adults from the Singapore Chinese Eye Study (SCES); (ii) Chinese children from the Singapore cohort Study of the Risk factors for Myopia (SCORM); and (iii) Malay adults from the Singapore Malay Eye Study (SiMES). SNPs that have been identified from this meta-analysis to be significantly associated with AL were further assessed for association with high myopia in an additional two independent case-control studies from Japan. We also examined the expression patterns of the candidate genes located in the vicinity of the identified SNPs in human ocular tissues and in the eyes of myopic mice.

## Results

A genome-wide meta-analysis of three GWAS on AL was performed in the post quality control samples from SCES (n = 1,860), SCORM (n = 929) and SiMES (n = 2,155). Principal component analysis (PCA) of these samples with reference to the HapMap Phase 2 individuals showed that the two Chinese cohorts (SCES and SCORM) are indistinguishable with respect to samples of Han Chinese descent, and the differentiation from samples of Japanese descent is evident only on the fourth principal component (Figure S1). The SiMES Malays are genetically similar to the Chinese-descent samples relative to individuals with European or African ancestries. The distributions of AL measurements in the three cohorts were approximately Gaussian and the baseline characteristics are summarized in Table 1. The mean AL were 23.98 mm (SD = 1.39 mm), 24.10 mm (SD = 1.18 mm) and 23.57 mm (SD = 1.04 mm) for SCES, SCORM and SiMES respectively. Moderate to high correlations between AL and SE were observed (SCES/SCORM/SiMES; Pearson correlation coefficient r = −0.75, −0.76 and −0.62 respectively). The meta-analysis was performed on 456,634 SNPs present in all three studies, and the quantile-quantile (QQ) plots of the *P*-values showed only modest inflation of the test statistics in SCES and in the meta-analysis (genomic control inflation factor: *λ*_meta_ = 1.03; *λ*_SCES_ = 1.05; *λ*_SCORM_ = 1.00; *λ*_SiMES_ = 1.00, Figure S2).

**Table 1:** Characteristics of study participants in the five Asian cohorts.

Characteristics	SCES^c^	SCORM^c^	SiMES^c^	Japan Dataset 1^d^		Japan Dataset 2^e^	
				High myopia	Controls	High myopia	Controls
Individuals (n)	1,860	929	2,155	483	1,194	504	550
Male (%)	51.5	51.7	49.3	33.7	41.3	43.3	49.5
Age^a^ (yrs)	58.4 (9.5)	10.8 (0.8)	57.7 (13.9)	58.8 (13.2)	50.3 (15.9)	37.8 (11.9)	39.7 (12.6)
Range of age	[44,85]	[10], [12]	[40,80]	[14,91]	[20,79]	[12], [76]	[21], [75]
Height^a^ (cm)							
Male	168.5 (6.3)	144.8 (8.7)	165.5 (6.4)	NA^f^	NA	NA	NA
Female	156.7 (5.5)	145.5 (8.9)	152.3 (6.2)	NA	NA	NA	NA
Education levels^b^ (%)							
No formal education	21.2	3.1	18.0	NA	NA	NA	NA
Primary education	33.5	19.1	8.5	NA	NA	NA	NA
Secondary education	24.9	39.7	46.6	NA	NA	NA	NA
Polytechnic	13.1	18.1	19.7	NA	NA	NA	NA
University	7.3	20.0	7.2	NA	NA	NA	NA
Average AL^a^ (mm)	23.97 (1.39)	24.13 (1.18)	23.57 (1.04)	30.08 (1.38)	NA	27.83 (1.28)	NA
Range of AL	[20.64, 33.36]	[21.05,28.20]	[20.48, 31.11]	[28.00, 38.03]	NA	[24.25, 34.74]	NA
Average SE^a^ (diopter)	−0.77 (2.64)	−2.02 (2.26)	−0.05 (1.90)	−14.86 (4.28)	NA	−11.61 (2.22)	NA
Range of SE	[−15.40, 6.25]	[−11.09, 3.78]	[−17.46, 8.56]	[−42.00, −2.50]	NA	[−23.00, −9.25]	≥−3.0

a Data presented are means (standard deviation). AL, ocular axial length; SE, spherical equivalent.

b The education levels of the children in SOCRM was presented by the level of educational attainment of the father, as.

c GWAS cohorts. SCES, Singapore Chinese Eye Study; SCORM, Singapore Cohort study of the Risk factors for Myopia; SiMES, Singapore Malay Eye Study.

d For the Japan dataset 1, high myopia, AL≥28 mm for both eyes; controls, general healthy population.

e For the Japan dataset 2, high myopia, SE≤−9.0 D for either eye; controls, SE≥−3.0 D for both eyes.

f NA, data not available.

**Figure 1:**
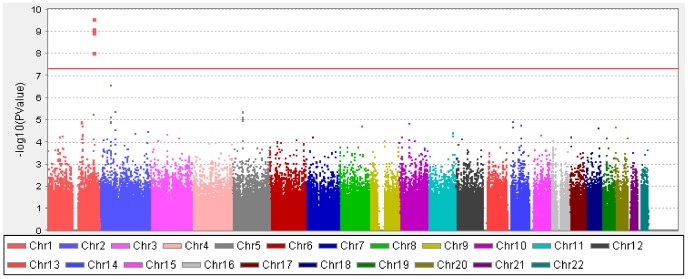
Manhattan plot of -log_10_(*P*) for the association on axial length from the meta-analysis in the combined cohorts of SCES, SCORM, and SiMES. The red horizontal line denotes genome-wide significance (*P* = 5×10^−8^).

A cluster of four SNPs on chromosome 1q41 (rs4373767, rs10779363, rs7544369 and rs4428898) attained genome-wide significance on meta-analysis for AL, adjusting for age, gender, height and education level (Figure 1). Analyses conducted without adjustment for height or education level yielded the same pattern of results. The most significant SNP rs4373767 (*P*_meta_ = 2.69×10^−10^) explained 0.98% of AL variance in SCES, 0.86% in SCORM and 0.73% in SiMES, and each copy of the minor allele (cytosine) decreased AL by 0.16 mm on average (Table 2). These top associated SNPs at chromosome 1q41 remained significant after adjustment for genomic control (*P*_meta_≤1.85×10^−8^). Table 2 also lists three genetic loci at chromosome 2p13.1 *(SEMA4F)*, 2p21 *(SPTBN1)* and 5q11.1 *(PARP8)* exhibiting suggestive evidence of association with AL that were seen in at least one SNP with *P*-values<1×10^−5^.

**Table 2:** Top SNPs (*P_meta_*-value≤1×10^−5^) associated with AL from the meta-analysis in the three Asian cohorts.

					SCES^c^ (n = 1,860)				SCORM^c^ (n = 929)		SiMES^c^ (n = 2,155)			Meta-analysis (n = 4,944)		
SNP	Nearest Gene	CHR	BP	MA^a^	MAF^b^	β^d^ (s.e)	*P*	MAF	β (s.e.)	*P*	MAF	β (s.e.)	*P*	β*_meta_* (s.e.)	*P_meta_*	*P_het_* e
**rs4373767**	ZC3H11B	1	217826305	C	0.30	−0.21 (0.05)	2.55 ×10^−6^	0.32	−0.16 (0.05)	1.80 ×10^−3^	0.24	−0.12 (0.04)	1.22 ×10^−3^	−0.16 (0.02)	2.69 ×10^−10^	0.23
**rs10779363**	ZC3H11B	1	217853513	C	0.29	−0.21 (0.05)	5.27 ×10^−6^	0.31	−0.17 (0.05)	1.63 ×10^−3^	0.24	−0.11 (0.04)	1.70 ×10^−3^	−0.15 (0.02)	7.83 ×10^−10^	0.23
**rs7544369**	ZC3H11B	1	217856085	T	0.29	−0.21 (0.05)	7.17 ×10^−6^	0.31	−0.16 (0.05)	2.56 ×10^−3^	0.24	−0.12 (0.04)	1.45 ×10^−3^	−0.15 (0.03)	1.10 ×10^−9^	0.29
**rs4428898**	ZC3H11B	1	217806589	G	0.30	−0.21 (0.05)	4.49 ×10^−6^	0.31	−0.14 (0.05)	7.46 ×10^−3^	0.22	−0.10 (0.04)	4.79 ×10^−3^	−0.14 (0.02)	9.07 ×10^−9^	0.19
rs4557020	SPTBN1	2	54571685	T	0.37	−0.11 (0.04)	9.08 ×10^−3^	0.37	−0.15 (0.05)	2.81 ×10^−3^	0.31	−0.11 (0.03)	8.10 ×10^−4^	−0.12 (0.02)	2.61 ×10^−7^	0.76
rs282544	PARP8	5	50062222	C	0.35	−0.10 (0.04)	2.38 ×10^−2^	0.33	−0.14 (0.05)	6.13 ×10^−3^	0.40	−0.09 (0.03)	2.40 ×10^−3^	−0.10 (0.02)	4.15 ×10^−6^	0.70
rs1137	SEMA4F	2	74792684	C	0.16	−0.01 (0.06)	8.68 ×10^−1^	0.18	0.22 (0.06)	5.97 ×10^−4^	0.26	0.14 (0.03)	3.01 ×10^−5^	0.12 (0.03)	4.26 ×10^−6^	0.02
rs2404958	PARP8	5	50098792	T	0.35	−0.10 (0.04)	2.91 ×10^−2^	0.33	−0.15 (0.05)	5.32 ×10^−3^	0.40	−0.09 (0.03)	2.46 ×10^−3^	−0.10 (0.02)	4.72 ×10^−6^	0.66
rs10735496	ZC3H11B	1	217790029	C	0.29	−0.14 (0.05)	1.95 ×10^−3^	0.30	−0.09 (0.05)	7.61 ×10^−2^	0.26	−0.10 (0.03)	3.66 ×10^−3^	−0.11 (0.02)	5.75 ×10^−6^	0.71
rs4671938	SPTBN1	2	54546708	G	0.35	−0.08 (0.04)	5.85 ×10^−2^	0.34	−0.12 (0.05)	1.61 ×10^−2^	0.33	−0.11 (0.03)	8.92 ×10^−4^	−0.10 (0.02)	7.46 ×10^−6^	0.82
rs32396	PARP8	5	50142196	A	0.35	−0.09 (0.04)	3.78 ×10^−2^	0.33	−0.14 (0.05)	6.98 ×10^−3^	0.40	−0.09 (0.03)	2.57 ×10^−3^	−0.10 (0.02)	7.73 ×10^−6^	0.69
rs12055210	PARP8	5	50025458	A	0.35	−0.10 (0.04)	2.38 ×10^−2^	0.33	−0.14 (0.05)	8.39 ×10^−3^	0.40	−0.09 (0.03)	4.01 ×10^−3^	−0.10 (0.02)	9.11 ×10^−6^	0.70
rs11954386	PARP8	5	50021409	A	0.35	−0.10 (0.04)	2.17 ×10^−2^	0.33	−0.14 (0.05)	6.34 ×10^−3^	0.40	−0.09 (0.03)	5.35 ×10^−3^	−0.10 (0.02)	9.44 ×10^−6^	0.63

a MA, minor allele.

b MAF, minor allele frequency in each cohort.

c GWAS cohorts. SCES - Singapore Chinese Eye Study; SCORM - Singapore Cohort study of the Risk factors for Myopia; SiMES - Singapore Malay Eye Study.

d β, coefficient of linear regression; s.e., standard error for coefficient β. Association between each genetic marker and AL was examined using linear regression, adjusted for age, gender, height and level of education. The effect sizes denote changes in millimeter of AL per each additional copy of the minor allele.

e *P_het_*, *P*-value for heterogeneity by Cochran's Q test across three study cohorts.

To assess whether these four SNPs at chromosome 1q41 have any role in high myopia predisposition, we performed association testing of these SNPs with high myopia in two independent case-control studies from Japan consisting of 987 high myopes and 1,744 controls. High myopes were defined as individuals with SE≤−9.00 D or AL≥28 mm (see Materials and Methods). All four SNPs exhibited consistent evidence of association (*P*<0.05) in both Japanese studies, suggesting a potential role of these SNPs for high myopia (Table 3).

**Table 3:** Association between genetic variants at chromosome 1q41 and high myopia in the five Asian cohorts.

			Japan Dataset 1 (483/1,194)^c^		Japan Dataset 2 (504/550)		SCES^b^ (44/1,305)		SCORM^b^ (65/332)		SiMES^b^ (22/2,052)		Meta–analysis (1,118/5,433)		
SNP	BP	MA^a^	OR^d^ (95% CI)	*P*	OR (95% CI)	*P*	OR (95% CI)	*P*	OR (95% CI)	*P*	OR (95% CI)	*P*	OR (95% CI)	*P_meta_*	*P_het_* e
rs4428898	217806589	G	0.74 (0.64, 0.87)	2.33×10^−4^	0.76 (0.64, 0.91)	2.15×10^−3^	0.73 (0.45, 1.19)	2.06×10^−1^	0.63 (0.40, 0.99)	4.89×10^−2^	0.72 (0.32, 1.64)	4.37×10^−1^	0.74 (0.66, 0.83)	7.86×10^−8^	0.96
rs4373767	217826305	C	0.74 (0.63, 0.86)	1.44×10^−4^	0.81 (0.68, 0.96)	1.80×10^−2^	0.73 (0.44, 1.18)	1.99×10^−1^	0.59 (0.38, 0.94)	2.59×10^−2^	0.77 (0.35, 1.69)	5.16×10^−1^	0.75 (0.68, 0.84)	4.38×10^−7^	0.75
rs10779363	217853513	C	0.74 (0.63, 0.87)	2.11×10^−4^	0.81 (0.68, 0.96)	1.41×10^−2^	0.68 (0.41, 1.13)	1.35×10^−1^	0.62 (0.39, 0.98)	4.13×10^−2^	0.76 (0.34, 1.68)	4.96×10^−1^	0.76 (0.68, 0.85)	7.81×10^−7^	0.82
rs7544369	217856085	T	0.75 (0.64, 0.88)	3.05×10^−4^	0.82 (0.69, 0.97)	2.32×10^−2^	0.67 (0.39, 1.15)	1.45×10^−1^	0.62 (0.39, 0.98)	4.17×10^−2^	0.74 (0.33, 1.64)	4.56×10^−1^	0.76 (0.69, 0.85)	1.45×10^−6^	0.79

a MA, minor allele.

b GWAS cohorts; SCES - Singapore Chinese Eye Study; SCORM - Singapore Cohort study of the Risk factors for Myopia; SiMES - Singapore Malay Eye Study.

c The sample sizes for each study denote the number of high-myopia cases versus controls. For the Japan dataset 1, high myopia, AL≥28mm for both eyes; controls, general healthy population; For the Japan dataset 2, SCES, and SiMES, high myopia, SE≤−9.0 D for either eye; controls, SE≥−3.0 D for both eyes; For SCORM children, high myopia, SE≤−6.0 D for either eye; controls, SE≥−1.0 D for both eyes.

d OR, odds ratio per copy of minor allele.

e *P_het_*, *P*-value for heterogeneity by Cochran's Q test across five study cohorts.

We further dichotomized the quantitative refraction from our three population-based studies (SCORM, SCES, and SIMES) to define samples as high myopes and controls according to similar criteria from the Japanese datasets. High myopes in SCES and SiMES were younger and more highly educated than controls (Table S1). While the case-control associations of these 4 SNPs with high myopia did not achieve statistical significance in SCES and SiMES, this is likely a consequence of the small sample sizes since the direction and magnitude of the odds ratios were highly similar across all cohorts. The meta-analysis of 1,118 high myopia cases and 5,433 controls from all the five cohorts yielded strong evidence of association with high myopia at these SNPs (*P*_meta_ between 1.45×10^−6^ to 7.86×10^−8^, Table 3), with no evidence of inter-study heterogeneity (*P*≥0.75 for heterogeneity). The minor allele cytosine at rs4373767 lowered the odds of high myopia by 25% with respect to the thymidine allele (OR_meta_ = 0.75, 95% CI: 0.68–0.84, *P*_meta_ = 4.38×10^−7^). The stringent definition of high myopia (SE≤−9.00D) used here only considered between 1.0% to 2.4% of our samples as cases, and relaxing this criterion to the commonly adopted threshold of SE≤−6.00D identified more myopia cases and increased the statistical support of all four SNPs (*P*_meta_ between 1.47×10^−7^ to 9.13×10^−9^, Table S2).

**Figure 2:**
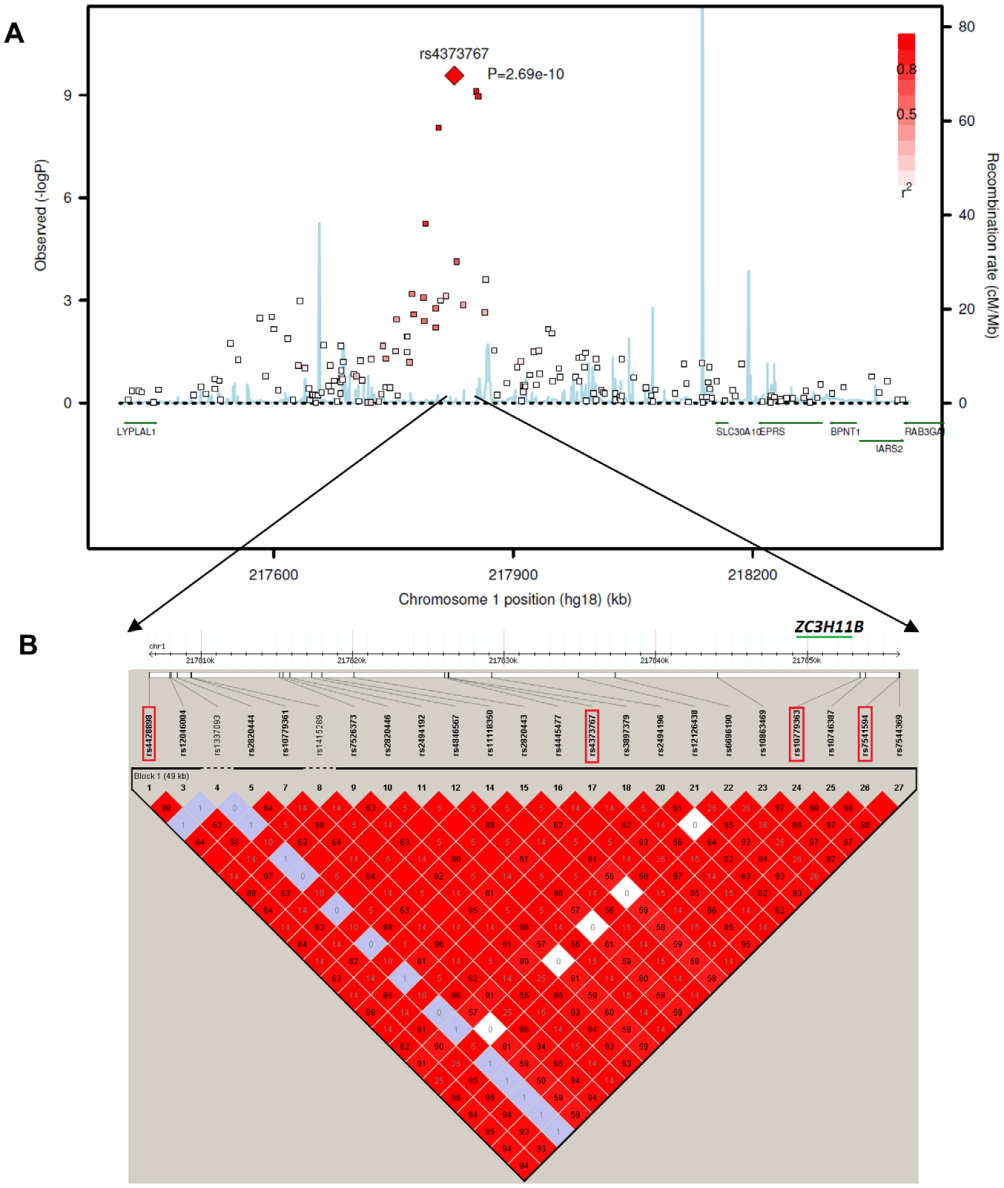
The chromosome 1q41 region and its association with axial length in the Asian cohorts. A) Regional plots for AL from the meta-analysis of three Asian GWAS cohorts: SCES, SCORM and SiMES. The association signals in a 1 megabase (Mb) region at chromosome 1q41 from 217,400 kb to 218,400 kb around the top SNP rs4373767 (red diamond) are plotted. The degree of pair-wise LD between the rs4373767 and any genotyped SNPs in this region is indicated by red shading, measured by r^2^. Superimposed on the plots are gene locations and recombination rates in HapMap Chinese and Japanese populations (blue lines). B) LD plot showing pair-wise r^2^ for all the SNPs genotyped in HapMap database residing between rs4428898 and rs7544369, inclusively, at chromosome 1q41. The four identified top SNPs are in red rectangles. The LD plot is generated by Haploview using SNPs (MAF>1%) genotyped on Han Chinese and Japanese samples in the HapMap database. All coordinates are in Build hg18.

This associated interval spans approximately 70 kb in the extended linkage disequilibrium (LD) block within an intergenic region on chromosome 1q41 (pairwise r^2^>0.5 with the most significant SNP rs4373767, Figure 2A). Zinc finger family CCCH-type 11B pseudogene *ZC3H11B* (RefSeq NG_007367.2) is embedded between the associated top SNPs rs4373767 and rs10779363 (Figure 2B). The most significant SNP rs4373767 is located 223 kb downstream from *SLC30A10* (RefSeq NM_018713.2), which is a member of solute carrier family 30, and 354 kb downstream of *LYPLAL1* (RefSeq NM_138794.3), encoding a lysophospholipase-like protein.

**Figure 3:**
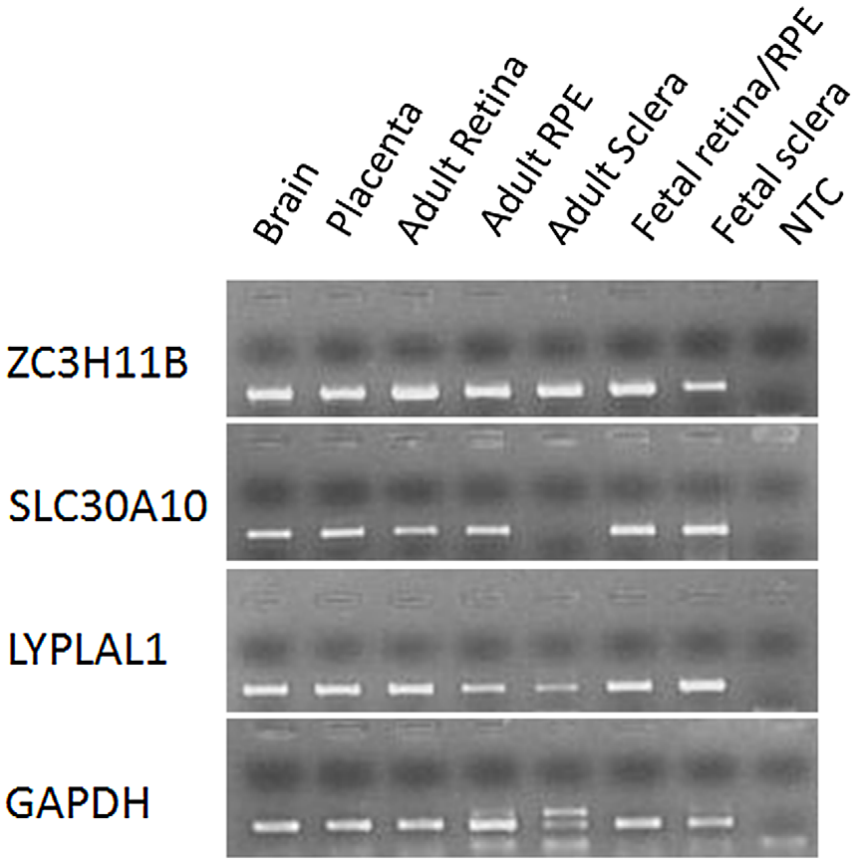
mRNA expression of *ZC3H11B, SLC30A10*, and *LYPLAL1* in human tissues. Expression of mRNA for the three genes was examined in human brain, placenta, neural retina (retina), retinal pigment epithelium (RPE) and sclera from adult tissues, and retina/RPE and sclera from 24-week gestation fetal tissues using reverse transcription polymerase chain reaction (RT-PCR). Glyceraldehyde 3-phosphate dehydrogenase (GAPDH) is a housekeeping gene and was used as an internal control for the quantification of mRNA expression. NTC (No template control) served as a negative control with the use of water rather than cDNA during PCR.

The mRNA expression levels of *ZC3H11B*, *SLC30A10* and *LYPLAL1* were surveyed in 24-week human fetal and adult tissues using reverse-transcriptase polymerase chain reaction (RT-PCR). Whilst *ZC3H11B* and *LYPLAL1* were found to be expressed across all the tissues including brain, placenta, neural retina, retina pigment epithelium (RPE) and sclera, the expression of *ZC3H11B* was more abundant compared to *LYPLAL1* (Figure 3). *SLC30A1*0 was expressed in all tissues but the adult sclera, analogous to observations made in other zinc transporters [21].

**Figure 4:**
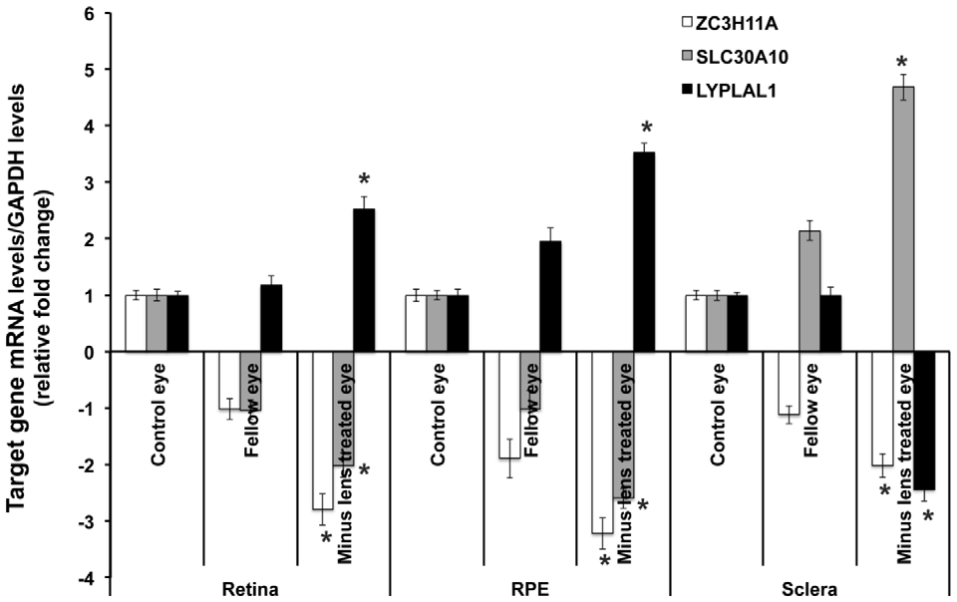
Transcription quantification of *ZC3H11A*, *SLC30A10*, and *LYPLAL1* in mouse retina, retinal pigment epithelium, and sclera in induced myopic eyes, fellow eyes, and independent control eyes. Myopia was induced using −15 diopter negative lenses in the right eye of mice for 6 weeks. Uncovered left eyes were served as fellow eyes and age-matched naive mice eyes were controls. Quantification of mRNA expression in mice neural retina (retina), retinal pigment epithelium (RPE) and sclera using quantitative real-time PCR. The bar represents the fold changes of mRNA for each gene after normalization using *GAPDH* as reference. The mRNA levels of murine *ZC3H11A*, a gene that is conserved with respect to *ZC3H11B* in human, *SLC30A10* and *LYPLAL1* in myopic and fellow retina, RPE and sclera are compared with independent controls with *P*-values as follows: *ZC3H11A* (retina/RPE/sclera, *P* = 2.60×10^−5^, 2.62×10^−6^ and 1.08×10^−4^ respectively), *SLC30A10* (*P* = 2.00×10^−4^, 2.00×10^−4^ and 4.02×10^−4^ respectively) and *LYPLAL1* (*P* = 1.50×10^−4^, 1.50×10^−4^, 1.54×10^−4^ respectively). *P<0.0001.

Gene expressions for *ZC3H11A*, *SLC30A10* and *LYPLAL1* from the tissues of myopic (with SE<−5.0 D) and fellow non-occluded eyes of the experimental mice were compared with age-matched control tissues (Figure 4). The mRNA levels of *ZC3H11A*, a gene that is conserved with respect to *ZC3H11B* in human, were significantly down-regulated in myopic eyes compared to naive controls (retina/RPE/sclera, Fold change = −2.88, −3.24 and −2.07; *P* = 2.60×10^−5^, 2.62×10^−6^ and 1.08×10^−4^, respectively). At the neighboring gene *SLC30A10*, there was a similarly significant reduction in the expression of mRNA in the retina tissue of myopic eyes in contrast to independent controls (retina/RPE, Fold change = −2.02, −2.69; *P* = 2.00×10^−4^, 2.00×10^−4^, respectively), with elevated expression in the sclera (Fold change = 4.58; *P* = 4.02×10^−4^). Another neighboring gene *LYPLAL1* exhibited up-regulation of transcription levels in retina tissue but was down-regulated in the sclera (retina/RPE/sclera, Fold change = 2.71, 3.45 and −2.36; *P* = 1.50×10^−4^, 1.50×10^−4^ and 1.54×10^−4^, respectively).

**Figure 5:**
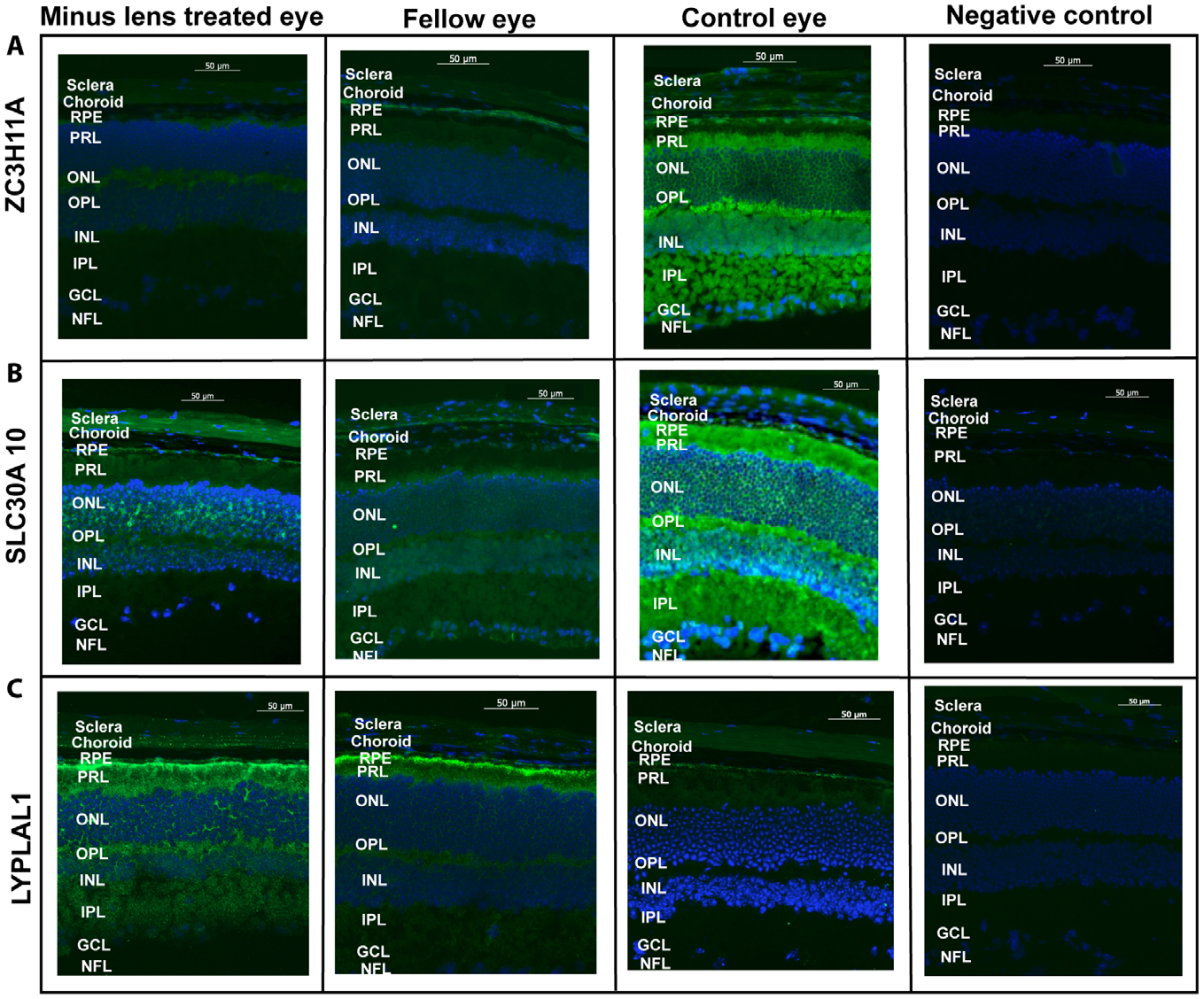
Immunofluorescent labeling. Immunofluorescent labeling of (A) *ZC3H11A* (B) *SLC30A10 and* (C) *LYPLAL1* in mouse retina, retinal pigment epithelium and sclera in induced myopic eyes, fellow eyes and independent control eyes. The neural retina (retina), retinal pigment epithelium (PRE) and scleral cells were immunolabeled with the polycolonal antibodies against *ZC3H11A*, *SLC30A10* and *LYPLAL1* and were co-labeled with 4′,6-diamidino-2-phenylindole (DAPI). Negative controls were devoid of a fluorescence signal, treated with the secondary antibody alone and DAPI. No immunostaining was observed in the negative controls. Scale bar represents 50 µM and magnification is 200×. The florescence intensity labeled of the green color shows the localization of proteins and blue color indicates the nuclei that were stained with DAPI. Expression of the proteins had a trend in abundance similarly to that of their mRNA levels as depicted in Figure 4. Lower level of expression was determined for *ZC3H11A* in all tissues for myopic mice. Similarly significant reduction was shown in the expression of *SLC30A10* in retina and RPE while higher level of expression was found in myopic sclera. *LYPLAL1* showed higher level of expression in the retina and RPE tissue but reduced expression in the sclera in myopic mice. The following abbreviations represent the retinal layers: nerve fibre layer (NFL), ganglion cell layer (GCL), inner plexiform layer (IPL), inner nuclear layer (INL), outer plexiform layer (OPL), outer nuclear layer (ONL), photo receptor layer (PRL) and retinal pigment epithelium (RPE).

Immunohistochemical results confirmed the localization of *ZC3H11A*, *SLC30A10* and *LYPLAL1* proteins in the neural retina, RPE and sclera (Figure 5). For *ZC3H11A*, positive immunostaining intensity was reduced significantly in the myopic tissues of experimental mice compared to the non-myopic independent controls (Figure 5A). This is consistent with the differential expression patterns at the transcription level. For *SLC30A10* and *LYPLAL1*, there were also similarly noticeable changes in the expression of proteins to that of their mRNA levels (Figure 5B and 5C).

## Discussion

We report that the chromosome 1q41 locus (most significant SNP rs4373767) is associated with AL in a meta-analysis of three GWAS performed in the study cohorts consisting of Chinese adults, Chinese children, and Malay adults. The discovery of chromosome 1q41 as a locus for high myopia in our data is further supported by validation in two independent Japanese cohorts, and the observed genetic effects are highly consistent across all five studies. The pseudogene *ZC3H11B* and two nearby genes *SLC30A10* and *LYPLAL1* were found to be expressed in the human retina and sclera. The potential roles in regulating myopia at three candidate genes were further implicated by the concordant changes in the pattern of transcription and protein expression in the mouse model.

The *ZC3H11B* pseudogene belongs to the CCCH-type zinc finger family, whereas such type of zinc finger protein has been shown as a RNA-binding motif to facilitate the mRNA processing at transcription [22]. Emerging evidence suggests that pseudogenes, resembling known genes but not producing proteins, play a significant role in pathological conditions by competing for binding sites to regulate the transcription of its protein-coding counterpart [23]–[25]. Although the function of the *ZC3H11B* in humans is presently unknown, the implicated role of the murine gene *ZC3H11A* (conserved gene of *ZC3H11B* in mouse) in myopia development is in keeping with previous findings that several zinc finger proteins are involved in myopia [26], [27]. Given their role as transcription factors [28], zinc finger protein *ZENK* has been proposed to function as a messenger in modulating the visual signaling cascade in the chicken retina, where the expression of the *ZENK* was suppressed by the condition of minus defocus (induced myopic eye growth) and enhanced by positive defocus (induced hyperopic eye growth) [29]–[31]. Similarly, it has been reported that *ZENK* knockout mice had elongated AL and a myopic shift in refraction [27]. Moreover, early growth response gene type1 *EGR-1* (the human homologue of *ZENK*) has been shown to activate transforming growth factor beta 1 gene *TGFB1* by binding its promoter [32], [33], a gene that is implicated to be associated with myopia [34], [35]. Another zinc protein finger protein 644 isoform *ZNF644* has recently been identified to be responsible for high myopia using whole genome exome sequencing in a Han Chinese family [26], whereas its influence on “myopia genes” remains to be elucidated. In light of this, the observation that ZC3H11B is abundantly expressed in retina and sclera, together with the significant down-regulation of the coding counterpart *ZC3H11A* in myopic mice eyes, suggests it may promote or inhibit the transcription of ocular growth genes vital in myopia development.

One of the two neighboring genes *SLC30A10* is an efflux transporter that reduces cytoplasmic zinc concentrations [36]. The *SLC30* zinc transporters are expressed abundantly in human RPE cells, and the retina has been observed to possess the highest concentration of zinc in the human body [21]. Zinc deficiency in the intracellular retina has thus been implicated in the pathogenesis of age-related macular degeneration (AMD) [37], [38], and in RPE-photoreceptor complex deficits, which can affect visual signal transduction from retina to sclera and lead to visual impairment [39]. *LYPLAL1* functions as a triglyceride lipase and this gene has been shown to be up-regulated in subcutaneous adipose tissue in obese individuals [40]–[42]. While the relationship between *LYPLAL1* and myopia is unknown, elevated saturated-fat intake has been proposed to influence myopia development through the retinoid receptor pathway [43]–[45]. Interestingly, the SNPs pinpointing chromosome 1q41 in our study are 1 Mb away from the transforming growth factor beta 2 gene (*TGFβ2*) which has been implicated in the down-regulation of mRNA levels in myopia progression of an induced tree shrew myopia model [46]. None of these nearby genes, however, are within the LD block containing our identified SNPs.

Chromosome 1q41 is a previously reported locus for refraction from a linkage analysis of 486 pedigrees in the Beaver Dam Eye Study, US [47]. Using microsatellite markers, Klein *et al* identified novel regions of linkage to SE on chromosome 1q41, whereas the peak spanned a broad region near Marker D1S2141 (multipoint *P*<1.9×10^−4^). This result however was not replicated in a subsequent genome-wide linkage scan for SE with denser SNP markers, partially due to varying information of linkage conveyed by SNPs versus microsatellites [48]. The identified variants at chromosome 1q41 in our study were noted to exhibit weaker, albeit still significant, association with SE in SCES and SCORM (rs4373767, SCES/SCORM: *P* = 3.54×10^−3^, 3.49×10^−2^, respectively; Table S3), but not in SiMES (3.51×10^−1^), which is consistent with the lower correlation of AL and SE seen in the SiMES data, partially from increasing lens opalescence in the Malay population [49], [50].

Our data have shown that genetic variants on chromosome 1q41 influence the physiological attribute of AL and are also associated with high myopia. Elongation of AL is the major underlying structural determinant of high myopia, mostly accompanied with prolate eyeballs and thinning of the sclera, macula and retina [4]. Thus, high myopia is also defined as AL of >26 mm in some studies [13], [51]. It is possible that genes involved in a quantitative trait (refraction or underlying AL) also play a role in the extreme forms of the trait (high myopia) [52]. Two recent GWAS performed in general Caucasians population have identified genetic variants for quantitative refraction at chromosome 15q14 [11] and 15q25 [12], of which the locus on 15q14 was subsequently confirmed to be associated with high myopia in the Japanese [53]. Our GWAS results herein highlight AL QTLs relevant for high myopia predisposition, which advances our understanding of the genetic etiology of myopia at different levels of severity.

The meta-analysis of three GWAS in our discovery suggests that the quantitative trait locus at chromosome 1q41 accounts for variation in AL in both school children and adults, regardless of age differences. Notably, the early-onset of myopia in childhood may continuously progress toward high myopia in later life, while adult-onset of myopia is usually in the low or moderate form [54],[55]. The significant association on chromosome 1q41 for high myopia in adults and children thus also implicates this locus identified for AL is likely to be associated with early-onset myopia.

The prevalence of myopia among Asian population is considerably higher than in Caucasians [1]. Although distinct genetic mechanisms governing myopia may exist for populations with different genetic backgrounds, we believe there are polymorphisms involved in refractive variation that are shared across populations. However, the allele frequencies of these identified SNPs vary across populations. For instance, the minor C allele of rs4373767 was a major allele in the HapMap Africans and Europeans with frequency of 0.92 and 0.62 respectively. Four distinct linkage disequilibrium (LD) blocks existed in 50 kb region encapsulating our top SNPs in the HapMap Africans, whereas high LD was observed for the Chinese, Malays and Japanese populations. Such heterogeneity may confer different statistical power and confound the transferability of the same variants across populations [56], [57]. In addition, we note that the variability in refraction attributed to AL may vary in different ethnic groups. For example, AL has been reported to account for a larger proportion of the variation in refraction in East-Asian children compared to their Caucasian counterparts [58], therefore the increased power of refraction may reflect more variation in factors other than pure elongation of AL in certain ethnic groups.

In conclusion, our findings suggest that common variants at chromosome 1q41 are associated with AL and high myopia in a pediatric and an adult cohort, the latter incorporating Chinese, Malay and Japanese populations. Further evaluation of causal variants and underlying pathway mechanisms may contribute to early identification of children at highest risk of developing myopia, and eventually lead to appropriate interventions to retard the progression of myopia.

## Materials and Methods

### Discovery cohorts

#### Singapore Chinese Eye Study (SCES)

SCES is an ongoing population-based cross-sectional survey of eye diseases in Chinese adults aged 40 to 80 years residing in the Southwestern part of Singapore. The study began in 2007 and a detailed description was published elsewhere [59]. In brief, a total of 2,226 residents in the Southwestern area of Singapore completed comprehensive ophthalmologic examinations, including visual acuity assessments, refraction, lens and retinal imaging, and slit lamp examinations. Genome-wide genotyping was performed in 1,952 individuals. Completed post quality control (QC) data for GWAS were available for 1,860 adults with AL measurements.

#### Singapore Cohort study of the Risk factors for Myopia (SCORM)

A total of 1,979 children in grades 1, 2, and 3 from three schools in Singapore were recruited from 1999 to 2001 [17]. The children were examined on their respective school premises annually by a team of eye care professionals. The GWAS was conducted in a subset of 1,116 Chinese children [14], [60]. The phenotype used in this study was based on the AL measured on the 4^th^ annual examination of the study (children at age 10 to 12 years). Complete post-filtering data on AL measurements and SNP data were available in 929 children.

#### Singapore Malay Eye Study (SiMES)

SiMES is a population-based cross-sectional survey of eye diseases in Malay adults aged 40 to 80 years living in Singapore. It was conducted between August of 2004 and June of 2006 [61]. A total of 4,168 Malay residents in the Southwestern area of Singapore were identified and invited for a detailed ocular examination where 3,280 (78.7%) participated. Genome-wide genotyping was performed in 3,072 individuals [62], [63]. Complete post-filtering data for GWAS with AL measurements were available for 2,155 subjects.

### Validation cohorts for high myopia

#### Japan dataset 1

The Japan dataset 1 consisted of 483 high myopia cases and 1,194 general healthy population controls. High myopia status was determined primarily on the basis of AL≥28 mm for both eyes, which corresponded to the spherical equivalent (SE) cut-off of at least −9.00 D [64]. Cases were recruited at the Center for Macular Disease of Kyoto University Hospital, the High Myopia Clinic of Tokyo Medical and Dental University, and the Fukushima Medical University Hospital. Details of the data have been reported elsewhere [13]. The population controls were recruited at the Aichi Cancer Center Research Institute.

#### Japan dataset 2

The Japan dataset 2 was comprised of 504 high myopia cases (SE≤−9.00 D in either eye) and 550 non-highly myopic controls (SE≥−3.00 D in both eyes). Less stringent thresholds were adopted for controls for the purpose of ease of recruitment from the clinics. Given the large phenotypic separation between the cases and controls, and assumption of homoscedasticity across genotype categories, such a study design using the extreme on one end (i.e. SE≤−9.00 D) but sampling less extreme controls (i.e. SE≥−3.00 D) still provides sufficient statistical power to detect the true positive signals in the association study [65]. Cases were recruited at the Yokohama City University and Okada Eye Clinic. Controls were obtained from the Yokohama City University and Tokai University Hospital.

### Measurements of AL, refractive error, and covariates

All the studies used a similar protocol for ocular phenotype measurements. For subjects in SCES and SiMES, AL for both eyes were measured using optical laser interferometry (IOLMaster V3.01, Carl Zeiss; Meditec AG Jena, Germany) [59], [61]. Children in the SCORM study underwent AL measurements using the A-scan ultrasound biometry machine (Echoscan US-800; Nidek Co, Tokyo, Japan) [17]. For subjects in the Japan dataset 1, applanation A-scan ultrasongraphy (UD-6000, Tomey, Nagoya, Japan) or partial coherence interferometry (IOLMaster, Carl Zeiss Meditec, Dublin, CA) were used to measure AL. AL was assessed using a portable A-scan Biometer/pachymeter (AL-2000, Tomey, Negoya, Japan) for the participants in the Japan dataset 2.

Non-cycloplegic refraction in SCES and SiMES as well as cycloplegic refraction in SCORM (three drops of 1% cyclopentolate at 5 minutes apart) were measured by autorefractor (Canon RK-5, Tokyo, Japan) [66]. For subjects in the Japan dataset 2, refraction was measured using auto-refraction ARK-730A (NIDEK), ARK-700A (NIDEK) and KR-8100P (TOPCON). SE was calculated as the sphere power plus half of the cylinder power for each eye.

To perform the genetic association of high myopia in SCES and SiMES, we used the definition adopted by the Japan case-control studies and defined high myopia cases as subjects having SE≤−9.0 D in at least one eye, and non high-myopia controls as samples with SE≥−3.0 D in both eyes. For children from SCORM aged 10 to 12 years, cases were defined as SE≤−6.0 D for at least one eye, while controls were defined as SE≥−1.0 D for both eyes; this is approximately equivalent to the projected SE of −9.0 and −3.0 respectively at university age based on the estimated annual progression rate in SE of −0.6 D for Chinese myopic children and −0.3 D in the controls [67]. Given the small sample sizes of high myopia cases identified in our population-based cohorts, in the supplementary analysis, we further applied the commonly adopted criteria of SE≤−6.0 D in either eye as cases. Controls were defined as SE≥−1.0 D in both eyes. For SCORM children, we retained the same criteria in both analyses. The detailed definitions of cases and controls are described in Table S4.

Age, gender, height and level of education were obtained from all Singapore participants who underwent ophthalmologic examination. Education was measured on an ordinal scale from no formal education to the highest educational level. For participants in SCORM, the education of the child was defined by the level of educational attainment of the father, as a marker of socioeconomic status.

### Ethics

All studies followed the principle of the Declaration of Helsinki. Study procedures and protocols were approved by the Institutional Review Board of each local institution involved in the study. In all cohorts, participants provided written, informed consent at the recruitment into the studies. Informed written consent was obtained from adult participants, and from the parents of the SCORM children.

Animal study approval was obtained from the SingHealth IACUC (AAALAC accredited). All procedures performed in this study complied with the Association of Research in Vision and Ophthalmology (ARVO) Statement for the Use of Animals in Ophthalmology and Vision Research.

### Genotyping and data quality control in discovery cohorts

For SCES, a total of 1,952 venous blood-derived samples were genotyped using Illumina Human 610 Quad Beadchips (Illumina Inc., San Diego, US) according to the manufacturer's protocols. Samples which failed genotyping or with low call rate (<95%, n = 11), with excessive heterozygosity (defined as sample heterozygosity exceeding 3 standard deviations from the mean sample heterogzygosity; n = 3), with gender discrepancies (n = 2) were excluded, as were cryptically related samples identified by the identity-by-state (IBS) (n = 41) and population structure in the principal components analyses (PCA) (n = 6). The criteria to define cryptically related samples and outliers with population structure in the discovery cohorts are described in the following paragraph. After the removal of the samples, SNP QC was then applied on a total of 579,999 autosomal SNPs for the 1,889 post-QC samples. SNPs were excluded based on (i) high rates of missingness (>5%) (n = 26,437); (ii) monomorphism or minor allele frequency (MAF)<1% (n = 59,633); or (iii) genotype frequencies deviating from Hardy-Weinberg Equilibrium (HWE) defined as HWE *P*-value<10^−6^ (n = 1,821). This yielded 492,108 autosomal SNPs. Those individuals with missing data on phenotypes were further removed (n = 29). Finally, 492,108 SNPs in 1,860 samples were available for analyses.

For SCORM, 1,116 DNA samples (1,037 from buccal swab and 79 from saliva) were genotyped on the Illumina HumanHap 550 Beadchips and 550 Duo Beadarrays. A total of 108 samples were excluded, comprising (i) 70 samples with call rates below 98%; (ii) 6 with poor genotyping quality; (iii) 11 samples identified from sib-ships; (iv) 18 with inconsistent gender information; and (v) 3 due to population structure. This left a total of 1,008 samples for further SNP QC. Based on 514,849 autosomal SNPs, we excluded 32,669 markers if they had missing genotype calls >5%, MAF<1%, or significantly deviated from HWE (*P*<10^−6^) [14]. A final set of 929 samples with 482,180 post-QC SNPs and completed AL measurement were included in analyses.

For SiMES, 3,072 DNA samples were genotyped using the Illumina Human 610 Quad Beadchips. The detailed QC procedures were provided elsewhere [68]. In brief, we omitted a total of 530 individuals due to: (i) subpopulation structure (n = 170); (ii) cryptic relatedness (n = 279); (iii) excessive heterozygosity or high missingness rate >5% (n = 37); and (iv) gender discrepancy (n = 44). After the removal of the samples, SNP QC was then applied on a total of 579,999 autosomal SNPs for the 2,542 post-QC samples. SNPs were excluded based on: (i) high rates of missingness (>5%) (n = 26,343); (ii) monomorphism or MAF<1% (n = 34,891); or (iii) genotype frequencies deviating from HWE (*P*<10^−6^) (n = 3,645). This yielded 515,120 SNPs after the same SNP QC criteria. Individuals without valid measurements for AL were further removed (n = 387). After the above filtering criteria, 515,120 SNPs in 2,155 samples were available for association analyses.

In our discovery cohorts, IBS was estimated with the genome-wide SNP data using PLINK software to assess the degree of recent shared ancestry for a pair of individuals [69]. For a pair of putatively-related samples defined as an identity by descent (IBD) value greater than 0.185 [70], we removed one individual from each pair of monzygotic twins/duplicates, parent-offspring or full-siblings etc. Population structure was ascertained using PCA with the EIGENSTRAT program and genetic outliers were defined as individuals whose ancestry was at least 6 standard deviations from the mean on one of the top ten inferred axes of variation [71].

For SiMES Malays, we also excluded the samples falling in the main clusters of PCA plots of the Chinese and Indians ethnic groups, as described in the previous study [68]. In SiMES, we noticed some degree of admixture in genetic ancestry of Malays and thus adjusted for ancestry along the top five axes of variation, as the spread of principal component scores was greater for the top five eigenvectors in the bivariate plots of PCA (Figure S3), The top ten principal components explained a small percentage of the global genetic variability of 1.3% while top five explained 1.0%, suggesting, all together, they had minimal effects on our association analyses.

### Validation cohorts for high myopia

High myopia cases in the Japan dataset 1 were genotyped using Illumina Human-Hap550 and 660 chips [13], while controls in the Japan dataset 1 were genotyped on Illumina Human-Hap610 chips. Subjects in the Japan dataset 2 were genotyped on the Affymetrix GeneChip Human Mapping 500 K Array Set (Affymetrix Inc., Santa Clara, US). For SNPs not available on the Affymetric chips (rs43737678, rs10779363 and rs7544369), genotyping was performed with TaqMan 5′ exonuclease assays using primers supplied by Applied Biosystems (Foster City, US). The probe fluorescence signal was detected using the TaqMan Assay for Real-Time PCR (7500 Fast Real-Time PCR System, Applied Biosystems).

### Gene expression in a mouse model of myopia

Experimental myopia was induced in B6 wild-type (WT) mice (n = 36) by applying a −15.00 D spectacle lens on the right eye (experimental eye) for 6 weeks since post-natal day 10. The left eyes were uncovered and served as contra-lateral fellow eyes. Age matched naive mice eyes were used as independent control eyes (n = 36). Each eye was refracted weekly using the automated infrared photorefractor as described previously [72]. AL was measured by AC- Master, Optic low coherence interferometry (Carl-Zeiss), in-vivo at 2, 4 and 6 weeks after the induction of myopia [73]. The minus-lens-induced eyes after six weeks were significantly associated with increased AL and myopic shift in refraction of <−5.00 D as compared to independent control eyes (n = 36, *P* = 3.00×10^−6^ for AL, and 2.05×10^−4^ for refraction). Eye tissues were collected at 6 weeks post myopia induction for further analyses.

Total RNA was isolated from pooled cryogenically ground mouse neural retina (retina), retinal pigment epithelium (RPE) and sclera for three batches using TRIzol Reagent (Invitrogen, Carlsbad, CA) with each batch (n = 6) comprising the myopic eye, fellow eye and control eye. RNA concentration and quality were assessed by the absorbance at 260 nm and the ratio of absorbance ratio at 260 and 280 nm respectively, using Nanodrop ND-1000 Spectrophotometer (Nanodrop Technologies, Wilmington, DE). RNA was purified using the RNeasy Mini kit (Qiagen, GmbH).

500 ng of purifed RNA was reverse-transcribed into cDNA using random primers and reagents from iScriptTM select cDNA synthesis kit (Bio-rad Laboratories, Hercules, CA). The pseudogene *ZC3H11B* (zinc finger CCCH type containing 11B) is not characterized in the mouse genome, therefore we examined a similar gene *ZC3H11A* (zinc finger CCCH type containing 11A) in mice. *ZC3H11A* in mice and *ZC3H11B* in humans are highly conserved with 79% nucleotide similarity by BLAST alignment analysis (http://blast.ncbi.nlm.nih.gov). We used quantitative Real-Time PCR (qRT-PCR) to validate the gene expression. qRT-PCR primers (Table S5) were designed using ProbeFinder 2.45 (Roche Applied Science, Indianapolis, IN) and this was performed using a Lightcycler 480 Probe Master (Roche Applied Science, Indianapolis, IN). The reaction was run in a Lightcycler 480 for 45 cycles under the following conditions: 95°C for 10 s, 56°C for 10 s and 72°C for 30 s. Gene expressions in the retina, RPE and sclera after six weeks of myopic eyes and the fellow eyes were compared to the control eyes. Glyceraldehyde 3-phosphate dehydrogenase (GAPDH) was used as an endogenous internal control.

### Immunohistochemistry

Whole mouse eyes (6 weeks minus lens treated myopic, contra-lateral fellow and independent control eyes, n = 6 per type) were embedded in frozen tissue matrix compound at −20°C for 1 hour. Prepared tissue blocks were sectioned with a cryostat at 6 microns thicknesses and collected on clean polysine™ glass slides. Slides with the sections were air dried at room temperature (RT) for 1 hour and fixed with 4% para-formaldehyde for 10 min. After washing 3X with 1x PBS for 5 minutes, 4% bovine serum albumin (BSA) diluted with 1x PBS was added as a blocking buffer. The slides were then covered and incubated for 1 hour at RT in a humid chamber. After rinsing with 1x PBS, a specific primary antibody raised in rabbit against *ZC3H11A, SLC30A10* and raised in goat against *LYPLAL1* (Abcam, Cambridge, UK) diluted (1∶200) with 4% BSA was added and incubated further at 4°C in a humid chamber overnight. After washing 3X with 1x PBS for 10 min, fluorescein-labeled goat anti-rabbit secondary antibody (1∶800, Invitrogen-Molecular Probes, Eugene, OR) and fluorescein-labeled rabbit anti-goat secondary antibody (1∶800, Santa Cruz Biotechnology, Inc. CA, USA) was applied respectively and incubated for 90 min at RT. After washing and air-drying, slides were mounted with antifade medium containing DAPI (4,6-diamidino-2-phenylindole; Vectashield, Vector Laboratories, Burlingame, CA) to visualize the cell nuclei. Sections incubated with 4% BSA and omitted primary antibody were used as a negative control. A fluorescence microscope (Axioplan 2; Carl Zeiss Meditec GmbH, Oberkochen, Germany) was used to examine the slides and capture images. Experiments were repeated in duplicates from three different samples.

### Gene expression in human tissues

*GAPDH*, *ZC3H11B, SLC30A10, and LYLPLAL1* were run using 10 ul reactions with Qiagen's PCR products consisting of 1.26 ul H_2_O, 1.0 ul 10X buffer, 1.0 ul dNTPs, 0.3 ul MgCl, 2.0 ul Q- Solution, 0.06 ul taq polymerase, 1.0 ul forward primer, 1.0 ul reverse primer and 1.5.0 ul cDNA. The reactions were run on a Eppendorf Mastercycler Pro S thermocycler with touchdown PCR ramping down 1°C per cycle from 72°C to 55°C followed by 50 cycles of 94°C for 0:30, 55°C for 0:30 and 72°C for 0:30 with a final elongation of 7:00 at 72°C. All primer sets were designed using Primer3 [74]. The gel electrophoresis was run on a 2% agarose gel at 70 volts for 35 minutes. The primers were run on a custom tissue panel including Clontech's Human MTC Panel I, Fetal MTC Panel I and an ocular tissue panel. The adult ocular samples were obtained from normal eyes of an 82-year-old Caucasian female from the North Carolina Eye Bank, Winston-Salem, North Carolina, USA. The fetal ocular samples were from 24-week fetal eyes obtained by Advanced Bioscience Resources Inc., Alameda, California, USA. All adult ocular samples were stored in Qiagen's RNA*later* within 6.5 hours of collection and shipped on ice overnight to the lab. Fetal eyes were preserved in RNA*later* within minutes of harvesting and shipped over night on ice. Whole globes were dissected on the arrival day. Isolated tissues were snap-frozen and stored at −80°C until RNA extraction. RNA was extracted from each tissue sample independently using the Ambion *mir*Vana total RNA extraction kit. The tissue samples were homogenized in Ambion lysis buffer using an Omni Bead Ruptor Tissue Homogenizer per protocol. Reverse transcription reactions were performed with Invitrogen SuperScript III First-Strand Synthesis kit.

### Statistical analysis

The primary analysis was performed on the AL quantitative trait. As a strong correlation exists in AL measurements from both eyes (r>0.9), we used the mean AL across both eyes in the GWAS analysis, as was recommended in a review [75]. Linear regression was used to interrogate the association of each SNP with AL after adjusting for age, gender, height and level of education, under the assumption of an additive genetic effect where the genotypes of each SNP are coded numerically as 0, 1 and 2 for the number of minor alleles carried. In addition, for SiMES, the top five principal components of genetic ancestry from the EIGENSTRAT PCA were also included as covariates to account for the effects of population substructure as described in genotype QC section [60]. Association tests between each genetic marker and phenotype were carried out using PLINK software [69] (version 1.07). Analyses were also repeated without adjustment for education level or height for the purpose of comparison.

In the discovery phase, we conducted a meta-analysis of GWAS results from 3 cohorts for AL using a weighted-inverse variance approach by fixed-effect modeling in METAL (http://www.sph.umich.edu/csg/abecasis/metal). In the secondary analyses, SNPs that have been identified from the primary analyses were tested for association with high myopia onset (as a binary trait) and SE (as a quantitative trait). For Singapore cohorts, the association analyses adjusted for the same covariates as the primary analyses within a linear regression and logistic regression framework respectively. For Japan case-control datasets, only age and gender were included as covariates in the model for high myopia, as the other covariates were not available.

The regional association plots were constructed by SNAP (http://www.broadinstitute.org/mpg/snap). Haploview 4.1 (http://www.broad.mit.edu/mpg/haploview) was used to visualize the LD of the genomic regions. Genotyping quality of all reported SNPs has been visually evaluated by the intensity clusterplots. The coordinates reported in this paper are on NCB136 (hg18).

For functional studies in the myopic mouse model, gene expression of all three identified genes in control and experimental groups was quantified using the 2^−ΔΔCt^ method [76]. The standard student's t-test was performed to determine the significance of the relative fold change of mRNA between the myopic eyes of the experimental mice with the independent age-matched controls.

## Supporting Information

Figure S1 Principal Component Analysis (PCA) of discovery cohorts SCES, SCORM and SiMES with respect to the four population panels in phase 2 of the HapMap samples (CEU - European, YRI – African, CHB – Chinese, JPT – Japanese) (A), and with respect to two reference population panels CHB and JPT (B–D). (A) Principal components 1 versus 2; the principal components (PCs) were calculated with SCES, SCORM, SiMES and four HapMap panels on the thinned set of 102,122 SNPs (r^2^<0.2). (B) Principal components 1 versus 2; (C) Principal components 1 versus 3; (D) Principal components 1 versus 4. For (B–D), the PCs were calculated with SCES, SCORM, SiMES and HapMap Asian population panels on the thinned set of 86,516 SNPs (r^2^<0.2).
(PDF)

Figure S2 Quantile-Quantile (Q-Q) plots of *P*-values for association between all SNPs and AL in the individual cohort (A) SCES, (B) SCORM, (C) SiMES, and combined meta-analysis of the discovery cohorts (D) SCES+SCORM+SiMES.
(PDF)

Figure S3 Principal Component Analysis (PCA) was performed in SiMES to assess the extent of population structure. Each figure represents a bivariate plot of two principal components from the PCA of genetic diversity within SiMES on the thinned set of 83,585 SNPs (r^2^<0.2). The first 5 principal components were used as covariates to account for population structure.
(PDF)

Table S1 Characteristics of high myopia cases and controls in three Singapore cohorts.
(DOCX)

Table S2 Association between genetic variants at chromosome 1q41 and high myopia in the meta-analysis of five cohorts.
(DOCX)

Table S3 Association between genetic variants at chromosome 1q41 and spherical equivalent (SE) in the meta-analysis of three Asian cohorts.
(DOCX)

Table S4 Definitions and numbers of high-myopia cases and controls used in the main and supplementary association analyses for high myopia.
(DOCX)

Table S5 Gene accession number in the nucleotide sequence database (NCBI), and qRT-PCR primer sequences in mice genome.
(DOCX)
