# Supplementary figures and images for: Genetic Variants on Chromosome 1q41 Influence Ocular Axial Length and High Myopia
Source: PLoS Genet. 2012 Jun 7;8(6):e1002753. doi: 10.1371/journal.pgen.1002753 (PMC3369958; doi:10.1371/journal.pgen.1002753)

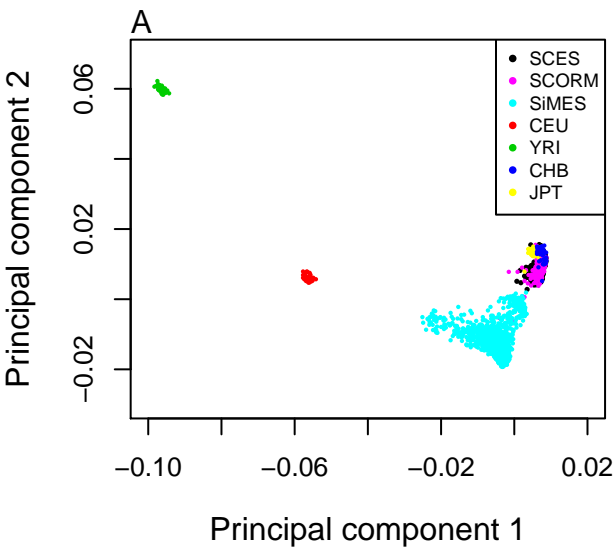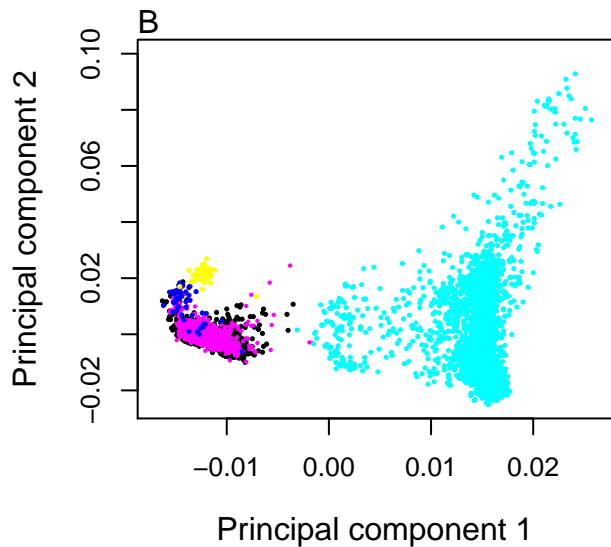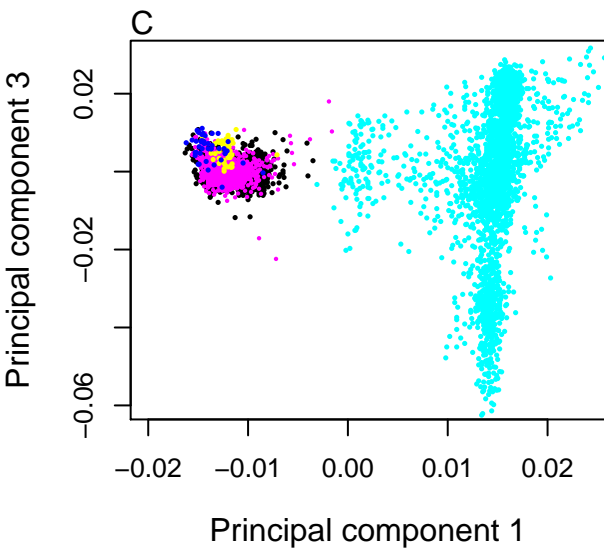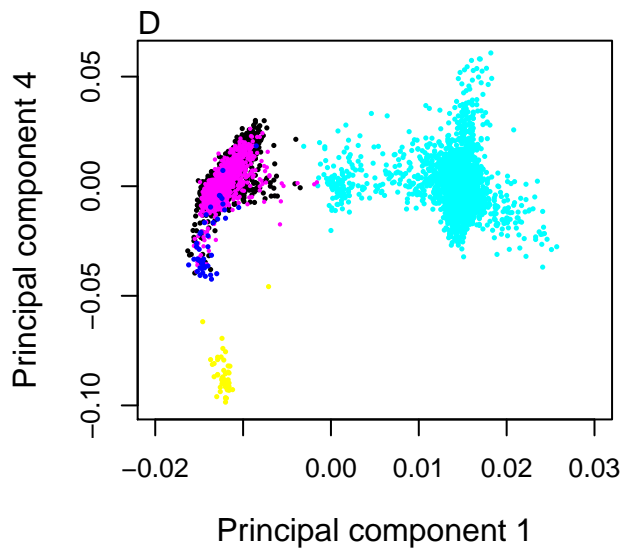

Supplement: Figure S1 — Principal Component Analysis (PCA) of discovery cohorts SCES, SCORM and SiMES with respect to the four population panels in phase 2 of the HapMap samples (CEU - European, YRI – African, CHB – Chinese, JPT – Japanese) (A), and with respect to two reference population panels CHB and JPT (B–D). (A) Principal components 1 versus 2; the principal components (PCs) were calculated with SCES, SCORM, SiMES and four HapMap panels on the thinned set of 102,122 SNPs (r2<0.2). (B) Principal components 1 versus 2; (C) Principal components 1 versus 3; (D) Principal components 1 versus 4. For (B–D), the PCs were calculated with SCES, SCORM, SiMES and HapMap Asian population panels on the thinned set of 86,516 SNPs (r2<0.2). (PDF) [file pgen.1002753.s001.pdf]

**A**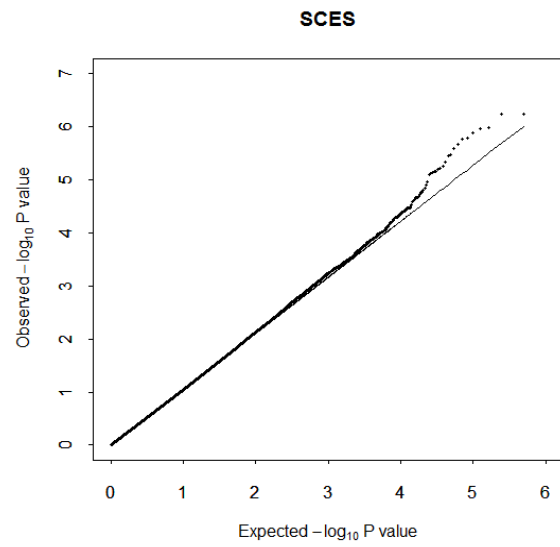**B**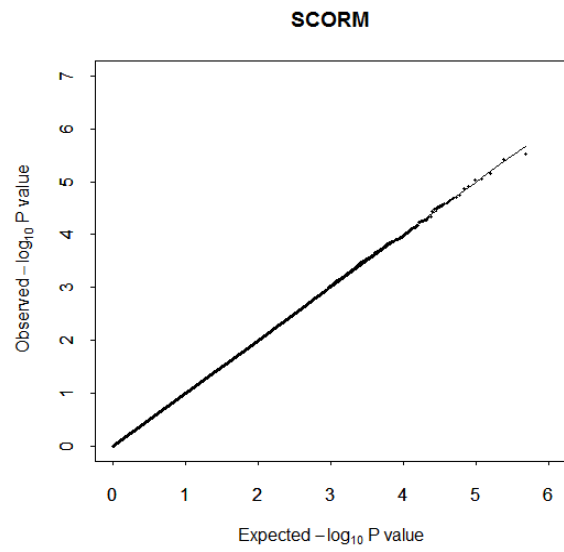**C**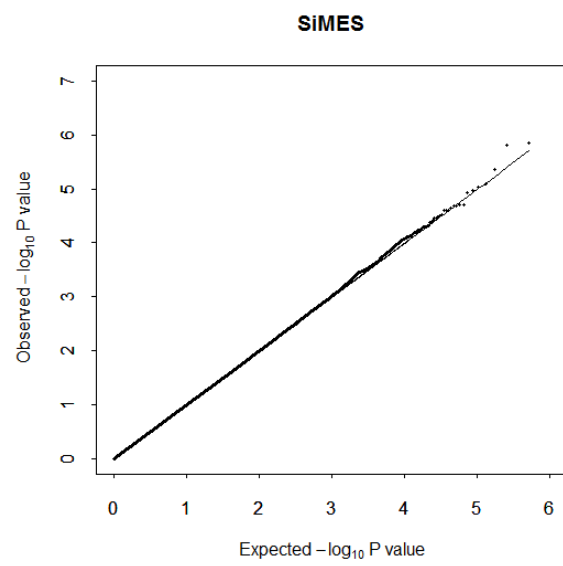**D**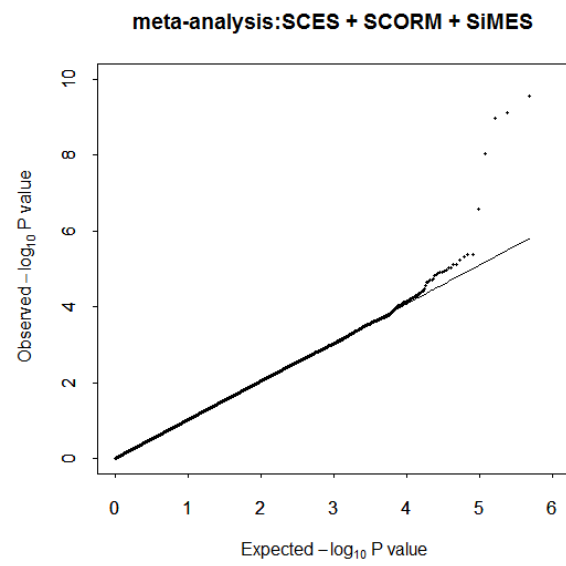

Supplement: Figure S2 — Quantile-Quantile (Q-Q) plots of P-values for association between all SNPs and AL in the individual cohort (A) SCES, (B) SCORM, (C) SiMES, and combined meta-analysis of the discovery cohorts (D) SCES+SCORM+SiMES. (PDF) [file pgen.1002753.s002.pdf]

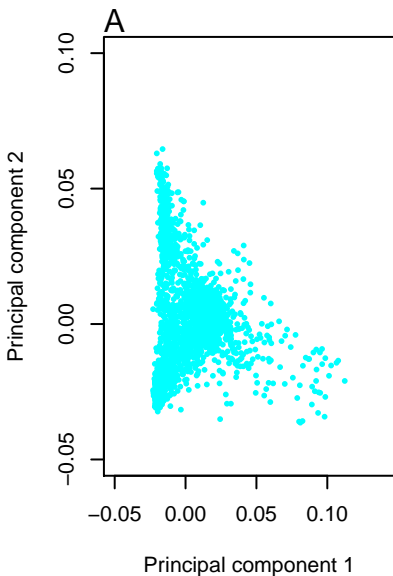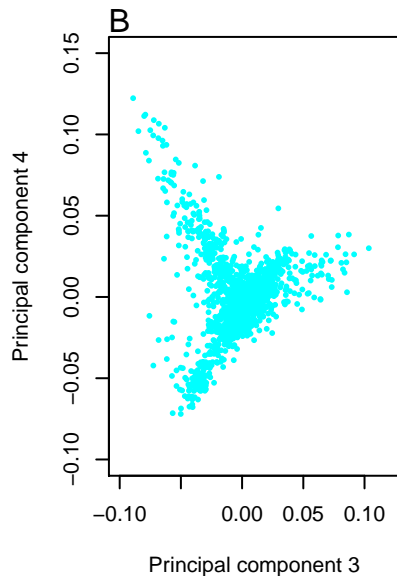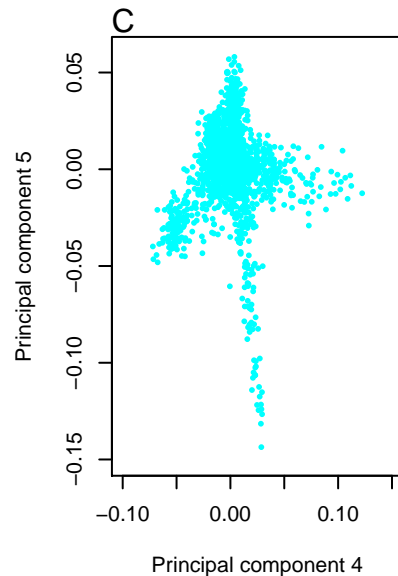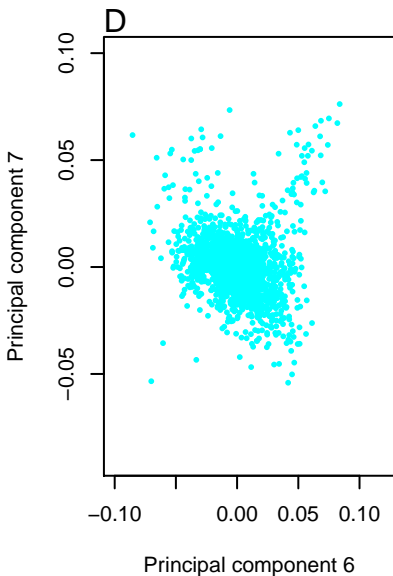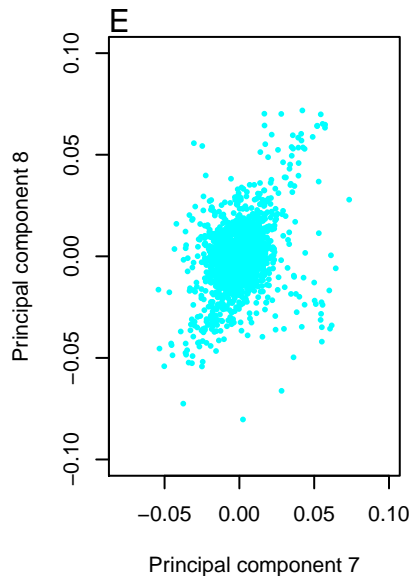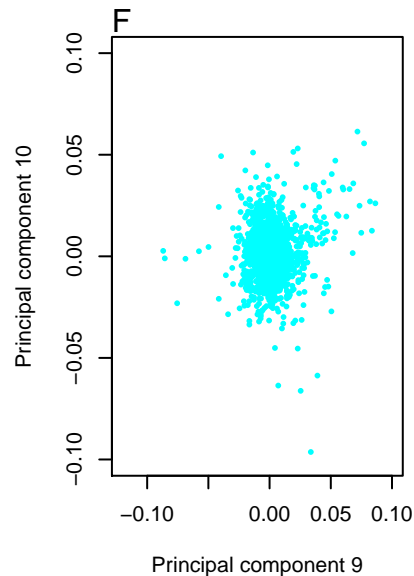

Supplement: Figure S3 — Principal Component Analysis (PCA) was performed in SiMES to assess the extent of population structure. Each figure represents a bivariate plot of two principal components from the PCA of genetic diversity within SiMES on the thinned set of 83,585 SNPs (r2<0.2). The first 5 principal components were used as covariates to account for population structure. (PDF) [file pgen.1002753.s003.pdf]
