# Supplementary material for: Genetic Variants on Chromosome 1q41 Influence Ocular Axial Length and High Myopia
Source: PLoS Genet. 2012 Jun 7;8(6):e1002753. doi: 10.1371/journal.pgen.1002753 (PMC3369958; doi:10.1371/journal.pgen.1002753)
Supplement: Table S1 — Characteristics of high myopia cases and controls in three Singapore cohorts. (DOCX) [file pgen.1002753.s004.docx]

**Table S1. Characteristics of high myopia cases and controls in three Singapore cohorts.**

| **Characteristics** | **SCES^c^** | | **SCORM** | | **SiMES** | |
| --- | --- | --- | --- | --- | --- | --- |
|  | High myopia | Controls | High myopia | Controls | High myopia | Controls |
| Individuals (n) | 45 | 1,305 | 65 | 332 | 22 | 2,052 |
| Male (%) | 46.7 | 50.5 | 61.5 | 52.4 | 55.6 | 49.8 |
| Age ^a^ (yrs) | 53.2 (8.7) | 57.4 (8.7) | 11.2 (0.9) | 10.8 (0.8) | 55.2 (12.7) | 57.6 (10.5) |
| Range of age | [45, 75] | [44, 84] | [10, 12] | [10, 12] | [40, 79] | [40, 80] |
| Height ^a^ (cm) |  |  |  |  |  |  |
| Male | 168.4 (7.7) | 168.9 (6.4) | 148.2 (9.2) | 145.0 (8.0) | 165.0 (8.1) | 165.6 [6.3] |
| Female | 158.9 (4.5) | 157.0 (5.5) | 149.2 (8.2) | 144.7 (9.0) | 151.2 (5.6) | 152.2 [6.2] |
| Education levels ^b^ (%) |  |  |  |  |  |  |
| No formal education | 13.6 | 19.6 | 1.5 | 5.7 | 22.2 | 18.0 |
| Primary education | 11.4 | 38.9 | 16.9 | 23.3 | 5.6 | 8.9 |
| Secondary education | 31.8 | 25.7 | 26.2 | 42.0 | 33.3 | 47.7 |
| Polytechnic | 36.4 | 11.0 | 27.7 | 14.8 | 16.7 | 19.0 |
| University | 6.8 | 4.8 | 27.7 | 14.2 | 22.2 | 6.4 |
| Average AL**^a^** (mm) | 27.46 (1.43) | 24.13 (1.18) | 25.97 (0.90) | 23.28 (0.77) | 27.84 (1.45) | 23.43 (0.82) |
| Range of AL | [25.24, 32.66] | [21.05,28.20] | [23.76, 28.20] | [21.05, 26.09] | [25.20,31.11] | [20.48 -26.52] |
| Average SE**^a^** (diopter) | -10.03 (2.25) | 0.24 (1.33) | -6.89 (1.06) | 0.20 (0.62) | -9.55 (2.92) | 0.35 (1.14) |
| Range of SE | [-15.40,-5.63] | [-3.0, 6.25] | [-11.09, -5.38] | [-0.93,3.78] | [-6.21,-17.46] | [-2.94, 8.56] |

^a^Data presented are means ( standard deviation). AL, ocular axial length; SE, spherical equivalent.

^b^ The education levels of the children in SOCRM was presented by the educational attainment of the father.

^c^GWAS cohorts. SCES, Singapore Chinese Eye Study; SCORM, Singapore Cohort study of the Risk factors for Myopia; SiMES, Singapore Malay Eye Study.

For SCES and SiMES, high myopia: SE ≤ -9.00 D for either eye; controls: SE ≥ -3.00 D for both eyes; For SCORM, high myopia: SE ≤ -6.00 D for either eye; controls: SE ≥ -1.00 D for both eyes.
