# Supplementary material for: Genetic Variants on Chromosome 1q41 Influence Ocular Axial Length and High Myopia
Source: PLoS Genet. 2012 Jun 7;8(6):e1002753. doi: 10.1371/journal.pgen.1002753 (PMC3369958; doi:10.1371/journal.pgen.1002753)
Supplement: Table S2 — Association between genetic variants at chromosome 1q41 and high myopia in the meta-analysis of five cohorts. (DOCX) [file pgen.1002753.s005.docx]

**Table S2.** Association between genetic variants at chromosome 1q41 and high myopia in the meta-analysis of five cohorts.

|  | | | **Japan dataset 1 (483/1,194)^a^** |  | **Japan dataset 2 (504/550)** |  | **SCES (115/1,040)** |  | **SCORM (65/332)** |  | **SiMES (60/1,713)** |  | **Meta -analysis** | |  |
| --- | --- | --- | --- | --- | --- | --- | --- | --- | --- | --- | --- | --- | --- | --- | --- |
| **SNP** | **BP** | **Minor Allele** | **OR^b^**  **(95% CI)** | ***P*** | **OR**  **(95% CI)** | ***P*** | **OR**  **(95% CI)** | ***P*** | **OR**  **(95% CI)** | ***P*** | **OR**  **(95% CI)** | ***P*** | **OR**  **(95% CI)** | ***P*** | ***P_het_*** |
| rs4428898 | 217806589 | G | 0.74  (0.64,0.87) | 2.33×10^-4^ | 0.76 (0.64,0.91) | 2.15×10^-3^ | 0.70  (0.50,0.98) | 3.49×10^-2^ | 0.63  (0.40,0.99) | 4.89×10^-2^ | 0.80 (0.50,1.30) | 3.73×10^-1^ | 0.74  (0.66,0.82) | 9.13×10^-9^ | 0.94 |
| rs4373767 | 217826305 | C | 0.74  (0.63,0.86) | 1.44×10^-4^ | 0.81  (0.68,0.96) | 1.80×10^-2^ | 0.69  (0.49,0.96) | 2.90×10^-2^ | 0.59 (0.38,0.94) | 2.59×10^-2^ | 0.87  (0.55,1.38) | 5.62×10^-1^ | 0.75  (0.68,0.84) | 6.14×10^-8^ | 0.64 |
| rs10779363 | 217853513 | C | 0.74  (0.63,0.87) | 2.11×10^-4^ | 0.81  (0.68,0.96) | 1.41×10^-2^ | 0.69  (0.49,0.97) | 3.35×10^-2^ | 0.62 (0.39,0.98) | 4.13×10^-2^ | 0.86 (0.54,1.36) | 5.14×10^-1^ | 0.76  (0.68,0.84) | 1.47×10^-7^ | 0.76 |
| rs7544369 | 217856085 | T | 0.75  (0.64,0.88) | 3.05×10^-4^ | 0.82  (0.69,0.97) | 2.32×10^-2^ | 0.68  (0.48,0.97) | 3.45×10^-2^ | 0.62 (0.39,0.98) | 4.17×10^-2^ | 0.83 (0.52,1.33) | 4.40×10^-1^ | 0.76  (0.69, 0.85) | 2.46×10^-7^ | 0.74 |

SCES - Singapore Chinese Eye Study; SCORM - Singapore Cohort study of the Risk factors for Myopia; SiMES - Singapore Malay Eye Study.

For SCES, SCORM and SiMES, high myopia cases was defined as SE ≤ -6.00 D for at least one eye; controls are samples with SE > -1.00 D in both eyes. For Japan cohorts, high myopia cases are defined as SE ≤ -9.00D (Japan dataset 2) or AL ≥ 28mm (Japan dataset 1).
^a^The sample size for each study denotes the number of high-myopia cases versus controls.

^b^OR, odds ratio per copy of minor allele.
