# Supplementary material for: Genetic Variants on Chromosome 1q41 Influence Ocular Axial Length and High Myopia
Source: PLoS Genet. 2012 Jun 7;8(6):e1002753. doi: 10.1371/journal.pgen.1002753 (PMC3369958; doi:10.1371/journal.pgen.1002753)
Supplement: Table S3 — Association between genetic variants at chromosome 1q41 and spherical equivalent (SE) in the meta-analysis of three Asian cohorts. (DOCX) [file pgen.1002753.s006.docx]

**Table S3.** Association between genetic variants at chromosome 1q41 and spherical equivalent (SE) in the meta-analysis of three Asian cohorts.

|  | | | |  | **SCES** |  |  | | **SCORM** | |  |  | | **SiMES** |  |  | **Meta-analysis** | |
| --- | --- | --- | --- | --- | --- | --- | --- | --- | --- | --- | --- | --- | --- | --- | --- | --- | --- | --- |
| **SNP** | **CHR** | **BP** | **Minor Allele** | **β** | **s.e.** | ***P*** | | **β** | **s.e.** | ***P*** | | | **β** | **s.e.** | ***P*** | **β** | **s.e.** | ***P_meta_*** |
| rs4428898 | 1 | 217806589 | G | 0.26 | 0.09 | 5.86×10^-3^ | | 0.18 | 0.11 | 9.38×10^-2^ | | | 0.08 | 0.07 | 2.66×10^-1^ | 0.15 | 0.05 | 2.32×10^-3^ |
| rs4373767 | 1 | 217826305 | C | 0.27 | 0.09 | 3.54×10^-3^ | | 0.23 | 0.11 | 3.49×10^-2^ | | | 0.07 | 0.07 | 3.51×10^-1^ | 0.16 | 0.05 | 1.43×10^-3^ |
| rs10779363 | 1 | 217853513 | C | 0.27 | 0.09 | 5.05×10^-3^ | | 0.21 | 0.11 | 6.22×10^-2^ | | | 0.08 | 0.07 | 2.32×10^-1^ | 0.16 | 0.05 | 1.44×10^-3^ |
| rs7544369 | 1 | 217856085 | T | 0.27 | 0.09 | 3.32×10^-3^ | | 0.21 | 0.11 | 6.86×10^-2^ | | | 0.07 | 0.07 | 2.91×10^-1^ | 0.16 | 0.05 | 1.56×10^-3^ |

SCES - Singapore Chinese Eye Study; SCORM-Singapore Cohort study of the Risk factors for Myopia; SiMES- Singapore Malay Eye Study.

β, coefficient of linear regression; s.e., standard error for coefficient β; Association between each genetic marker and SE was examined using linear regression, adjusted for age, gender, height and level of education. The effect sizes denote changes in diopters (D) per each additional copy of the minor allele.
