# Supplementary material for: Genetic Variants on Chromosome 1q41 Influence Ocular Axial Length and High Myopia
Source: PLoS Genet. 2012 Jun 7;8(6):e1002753. doi: 10.1371/journal.pgen.1002753 (PMC3369958; doi:10.1371/journal.pgen.1002753)
Supplement: Table S4 — Definitions and numbers of high-myopia cases and controls used in the main and supplementary association analyses for high myopia. (DOCX) [file pgen.1002753.s007.docx]

**Table S4**. Definitions and numbers of high-myopia cases and controls used in the main and supplementary association analyses for high myopia.

| Main analyses | **Japan dataset 1** | **Japan dataset 2** | **SCES** | **SCORM** | **SiMES** |
| --- | --- | --- | --- | --- | --- |
| # Cases /# Controls | 483/1,194 | 504/550 | 44/1,305 | 65/332 | 22/2,052 |
| Case definition | AL ≥ 28 mm (both eyes) | SE ≤ -9.00 D (either eye) | SE ≤ -9.00 D (either eye) | SE ≤ -6.00 D (either eye) | SE ≤ -9.00 D (either eye) |
| Control definition | General healthy population | SE ≥ -3.00 D (both eyes) | SE ≥ -3.0 D (both eyes) | SE ≥ -1.00 D (both eyes) | SE ≥ -3.00 D (both eyes) |
|  |  |  |  |  |  |
|  |  |  |  |  |  |
| Supplementary analyses | | | |  |  |
|  | **Japan dataset 1** | **Japan dataset 2** | **SCES** | **SCORM** | **SiMES** |
| # Cases / # Controls | 483/1,194 | 504/550 | 115/1,040 | 65/332 | 60/1,713 |
| Case definition | AL ≥ 28 mm (both eyes) | SE ≤ -9.00 D (either eye) | SE ≤ -6.00 D (either eye) | SE ≤ -6.00 D (either eye) | SE ≤ -6.00 D (either eye) |
| Control definition | General healthy population | SE ≥ -3.00 D (both eyes) | SE ≥ -1.00 D (both eyes) | SE ≥ -1.00 D (both eyes) | SE ≥ -1.00 D (both eyes) |

SCES -Singapore Chinese Eye Study; SCORM - Singapore Cohort study of the Risk factors for Myopia; SiMES - Singapore Malay Eye Study. SE, spherical equivalent in diopters (D); AL, ocular axial length in millimeter (mm).
