# Supplementary material for: Genetic Variants on Chromosome 1q41 Influence Ocular Axial Length and High Myopia
Source: PLoS Genet. 2012 Jun 7;8(6):e1002753. doi: 10.1371/journal.pgen.1002753 (PMC3369958; doi:10.1371/journal.pgen.1002753)
Supplement: Table S5 — Gene accession number in the nucleotide sequence database (NCBI), and qRT-PCR primer sequences in mice genome. (DOCX) [file pgen.1002753.s008.docx]

**Table S5.** Gene accession number in the nucleotide sequence database (NCBI), and qRT-PCR primer sequences in mice genome.

| **Gene** | **Accession number** | **Forward Primer** | **Reverse Primer** |
| --- | --- | --- | --- |
| *ZC3H11A* | NM_144530 | tttggcctgagaatttaccc | Tgtgcaaacagtttcatttcct |
| *SLC30A10* | NM_001033286 | gggcacagcagtgactctc | Ttctcggtgttcagggaatc |
| *LYPLAL1* | NM_146106 | cagtggattggttgatgaagaa | tcccatagaaaatccccctatt |
